# Applying spirometry phenotypes to a longitudinal cohort born very preterm

**DOI:** 10.1136/bmjresp-2025-004041

**Published:** 2026-06-04

**Authors:** Tiffany K Bradshaw, Sanja Stanojevic, Shannon J Simpson

**Affiliations:** 1 Wal-yan Respiratory Research Centre, Foundations of Lung Disease, The Kids Research Institute Australia, Perth, Western Australia, Australia; 2 School of Allied Health, Faculty of Health Sciences, Curtin University, Perth, Western Australia, Australia; 3 Department of Community Health and Epidemiology, Faculty of Medicine, Dalhousie University, Halifax, Nova Scotia, Canada

**Keywords:** Paediatric Lung Disease, Respiratory Function Test, Respiratory Measurement, Pulmonary Disease, Chronic Obstructive, Lung Physiology, Paediatric Physician

## Abstract

**Abstract:**

**Background:**

To better characterise prematurity-associated lung disease, adult spirometry phenotype classifications (obstructive lung disease, preserved ratio impaired spirometry and dysanapsis) have been applied to children born preterm. It is unknown how these phenotypes track over time.

**Aim:**

Apply spirometry phenotype classifications to a longitudinal cohort born very preterm (≤32 weeks gestation) and track changes in classifications from early childhood to adolescence.

**Methods:**

This retrospective longitudinal cohort study included children born very preterm in Western Australia between 1997 and 2003, followed up between 2007 and 2022. Spirometry testing and a modified International Study of Asthma and Allergies in Childhood questionnaire were completed at early childhood (4–8 years), middle childhood (9–12 years) and adolescence (16–23 years). Spirometry phenotype classifications were applied to each participant with acceptable spirometry at each time point.

**Results:**

200 participants completed 311 acceptable spirometry measurements. Abnormal spirometry phenotypes were observed in 29% of participants at early childhood, 41% at middle childhood and 43% at adolescence. Of those with spirometry measurements across at least two time points, 36% changed phenotypes (e.g. from normal to abnormal, from abnormal to normal or changed between the abnormal groups). Many of those that changed phenotype classification had a forced expiratory volume in 1 s (FEV_1_) z-score or FEV_1_ to forced vital capacity (FVC) ratio z-score sitting near the lower limits.

**Conclusion:**

It remains unclear whether changes in phenotype reflects the biological variability of the measure or disease progression. The instability of adult spirometry classifications across serial spirometry measurements suggests this approach does not adequately capture the complexity of prematurity-associated lung disease during the growth period.

**WHAT IS ALREADY KNOWN ON THIS TOPIC**

- Spirometry phenotypes have been applied to children born preterm to better characterise long-term lung function outcomes beyond the traditional bronchopulmonary dysplasia definition.

**WHAT THIS STUDY ADDS**

- This study found instability of spirometry phenotype classifications from early childhood to adolescence in a longitudinal cohort born very preterm, finding some individuals changing from a normal to abnormal phenotype, as well as abnormal to normal or between different abnormal groups.

**HOW THIS STUDY MIGHT AFFECT RESEARCH, PRACTICE OR POLICY**

- Though the instability of adult spirometry phenotype classification across serial spirometry measurements suggests this approach does not adequately capture the complexity of prematurity-associated lung disease during the growth period, this study stresses the importance of structured long-term respiratory follow-up of those born preterm.

## Introduction

 Preterm birth is a risk factor for impaired lung function, as measured by spirometry,^1^ and airway disease throughout life.^2^ Airflow obstruction may increase with increasing age,^1^ and some individuals born preterm are on a long-term trajectory towards chronic obstructive pulmonary disease (COPD).^2 3^ Prematurity and abnormal lung development is now recognised as a COPD aetiotype.^4^

In addition to classic airflow obstruction (forced expiratory volume in 1 s (FEV_1_) / forced vital capacity (FVC) < lower limit of normal (LLN); LLN=−1.645 z-score), reduced FEV_1_ with preserved FEV_1_/FVC ratio or Preserved Ratio Impaired Spirometry (PRISm)^5^ has been proposed in adult smokers as a precursor to COPD^6^ and is associated with increased risk of cardiopulmonary disease and premature mortality in adults.^5^ In addition, dysanapsis (reduced airway growth relative to lung volume) has also been established as a risk factor for COPD.^7^

A study by Cousins *et al*^8^ applied these adult spirometry phenotypes to 7–12 year-olds born <34 weeks gestation. They reported obstructive lung disease in 7.7%, PRISm in 9.0% and dysanaptic airway growth in 5.9% of preterm born children.^8^

In this study, we aimed to apply spirometry phenotype classifications to a cohort of children born very preterm (≤32 weeks gestation) and track how classifications changed between early childhood and adolescence.

## Methods

Participants were born ≤32 weeks gestation in Perth, Western Australia, between 1997 and 2003. Individuals with known major congenital or genetic abnormalities, including cardiac disease, cystic fibrosis and those with significant neurodevelopmental disability that prevented them from completing pulmonary function testing at the first visit, were excluded. Bronchopulmonary dysplasia (BPD) was defined as at least 28 days of supplemental oxygen requirement as assessed at 36 weeks’ post-menstrual age.^9^ This cohort was initially recruited 2:1 for preterm with BPD:preterm without BPD.^10^ Children were first recruited during early childhood (4–8 years), with the cohort further supplemented by additional recruitment at follow-up during middle childhood (9–12 years). All participants were invited for follow-up during adolescence (16–23 years).

Spirometry testing and a modified International Study of Asthma and Allergies in Childhood questionnaire^11^ to collect respiratory symptoms (wheeze, cough, rattle and/or shortness of breath at rest) in the 3 months prior to testing were completed at all three time points between 2007 and 2022 at Princess Margaret Hospital and Perth Children’s Hospital. Perinatal data were collected at time of enrolment from medical records. Spirometry measurements were completed according to contemporaneous technical standards at the time of testing, using the Sensormedics Vmax (Yorba Linda, California, USA) at time point 1 and 2 and the BodyBox 5500 (Medisoft, Sorinnes, Belgium) at time point 3. Acceptable and repeatable (European Respiratory Society (ERS)/American Thoracic Society (ATS) grade A or B) spirometry outcomes were expressed as z-scores according to the 2012 Global Lung Function Initiative equations^12^ for direct comparison to the study from Cousins *et al*.^8^ Parental and/or personal informed consent was obtained at each study visit. The protocol was approved by the Princess Margaret Hospital Human Ethics Committee (EP1760) and Child and Adolescent Health Service Human Research Ethics Committee (RGS815).

The spirometry classifications,^8^ prematurity-associated obstructive lung disease (POLD) (FEV_1_ and FEV_1_/FVC < LLN), prematurity-associated PRISm (pPRISm) (FEV_1_ < LLN and FEV_1_/FVC ≥ LLN), dysanapsis of prematurity (pDysanapsis) (FEV_1_ ≥ LLN and FEV_1_/FVC < LLN) and normal spirometry (FEV_1_ and FEV_1_/FVC ≥ LLN), were applied to each participant with acceptable spirometry at each time point.

Pearson’s χ^2^ test was used to compare proportions and a Cochran-Armitage test for trend was used to assess trend of classification into an abnormal phenotype across the three follow-ups. All analyses were performed using R V.4.5.0.

Patient and public involvement members were involved in prioritising this research. Neither patients nor the public were involved in the design, conduct or reporting of our research.

## Results

200 participants completed 408 lung function measurements and respiratory questionnaires over the three time points (early childhood, middle childhood and adolescence). Not all participants attended every follow-up or obtained acceptable and repeatable spirometry, resulting in 311 acceptable spirometry measurements available for this analysis, with 102 individuals having spirometry measurements across at least two time points. Demographics for each period are shown in table 1. The majority (57–71%) of participants were classified as normal at each age (table 1), with a greater percentage of ‘abnormal’ (POLD, pPRISm or pDysanapsis) at each follow-up point (29%, 41% and 43%, χ^2^ for trend, p=0.057).

**Table 1:** Demographics and spirometry phenotype classifications at each time point

Participants, N	200
Male, N (%)	120 (60%)
Ethnicity	
White	185 (93%)
Other	15 (7%)
Maternal and early life factors	
Maternal steroids, N (%)	162/192 (84%)
Smoking during pregnancy, N (%)	31/192 (16%)
Maternal asthma, N (%)	42/196 (21%)
Gestational age (weeks); median (IQR)	28.0 (25.6–29.9)
Birth weight (g); median (IQR)	975.0 (772.5–1312.5)
Birth weight z-score	−0.1±0.9
Bronchopulmonary dysplasia, N (%)*	126/200 (63%)
Received surfactant, N (%)	147/198 (74%)
Received postnatal steroids, N (%)	48/200 (24%)
Duration of NICU oxygen supplementation (days); median (IQR)	48.0 (3.0–92.5)
Duration of NICU mechanical ventilation (days); median (IQR)	4.0 (0.5–25.0)
Duration of NICU CPAP (days); median (IQR)	6.0 (1.0–18.0)
Life course factors	
Respiratory admission ever, N (%)	92/195 (47%)
Physician diagnosed asthma ever, N (%)	94/199 (47%)
Ever smoked, N (%)	6/128 (5%)
Visit 1: early childhood	
N	118
Male, N (%)	71 (60%)
Age (years); median (IQR)	6.1 (5.3–6.8)
Acceptable spirometry, N (%)	76 (64%)
FEV_1_ z-score	−0.5±1.1
FEV_1_/FVC z-score	−0.9±1.1
Spirometry phenotype classification:	
Normal spirometry	54 (71%)
POLD	6 (8%)
pPRISm	3 (4%)
pDysanapsis	13 (17%)
Missing	42
Visit 2: middle childhood	
N	163
Male, N (%)	99 (61%)
Age (years); median (IQR)	10.8 (10.3–11.3)
Acceptable spirometry, N (%)	122 (75%)
FEV_1_ z-score	−0.7±1.1
FEV_1_/FVC z-score	−1.3±1.0
Spirometry phenotype classification:	
Normal spirometry	72 (59%)
POLD	18 (15%)
pPRISm	5 (4%)
pDysanapsis	27 (22%)
Missing	41
Visit 3: adolescence	
N	127
Male, N (%)	71 (56%)
Age (years); median (IQR)	19.3 (18.4–20.4)
Acceptable spirometry, N (%)	113 (89%)
FEV_1_ z-score	−0.9±1.2
FEV_1_/FVC z-score	−1.2±1.1
Spirometry phenotype classification:	
Normal spirometry	64 (57%)
POLD	20 (18%)
pPRISm	7 (6%)
pDysanapsis	22 (19%)
Missing	14

* Participants were recruited 2:1 for preterm with bronchopulmonary dysplasia (BPD):preterm without BPD

† Data are reported mean±SD unless otherwise specified.

CPAP, continuous positive airway pressure; FEV_1_, forced expiratory volume in 1 s; FVC, forced vital capacity; NICU, neonatal intesive care unit; pDysanapsis, dysanapsis of prematurity; POLD, prematurity-associated obstructive lung disease; pPRISm, prematurity-associated preserved ratio impaired spirometry.

A similar proportion of those with a neonatal diagnosis of BPD compared with no-BPD were classified into an abnormal spirometry phenotype at early childhood (27% BPD (n/n=13/48) vs 32% no-BPD (n/n=9/28); p=0.836) and adolescence (50% BPD (n/n=36/72) vs 32% no-BPD (n/n=13/41); p=0.091), with an increased proportion of children with BPD belonging to an abnormal phenotype at middle childhood (52% BPD (n/n=38/73) vs 24% no-BPD (n/n=12/49); p=0.004).

Participants with respiratory symptoms were similarly assigned to abnormal spirometry and normal spirometry phenotypes at early childhood (75% (n/n=9/12) vs 72% (n/n=21/29); p=1), middle childhood (57% (n/n=27/47) vs 49% (n/n=34/69); p=0.499) and adolescence (79% (n/n=27/34) vs 86% (n/n=36/42); p=0.675).

### Longitudinal spirometry phenotype changes

**Figure 1:**
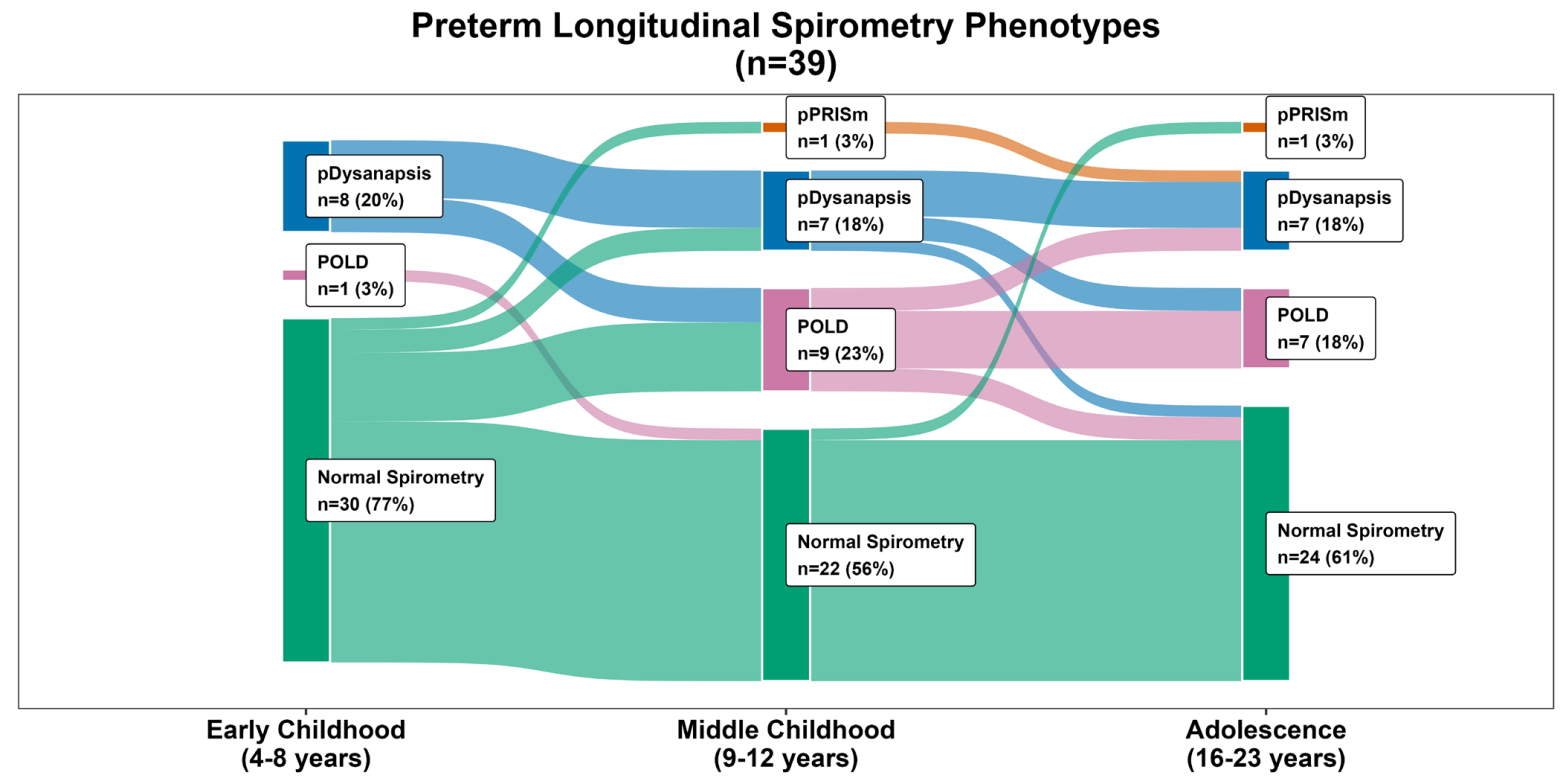
Spirometry phenotypes applied to a very preterm cohort from early childhood to adolescence. pDysanapsis, dysanapsis of prematurity; POLD, prematurity-associated obstructive lung disease; pPRISm, prematurity-associated preserved ratio impaired spirometry.

Acceptable and repeatable spirometry was obtained at all three time points by 39 participants (figure 1). Of these, 22 individuals (56%) were classified into the same spirometry phenotype throughout, and 17 (44%) individuals changed phenotypic grouping in both directions (eg, from abnormal to normal, from normal to abnormal and changed between the abnormal groups) (figure 1).

**Figure 2:**
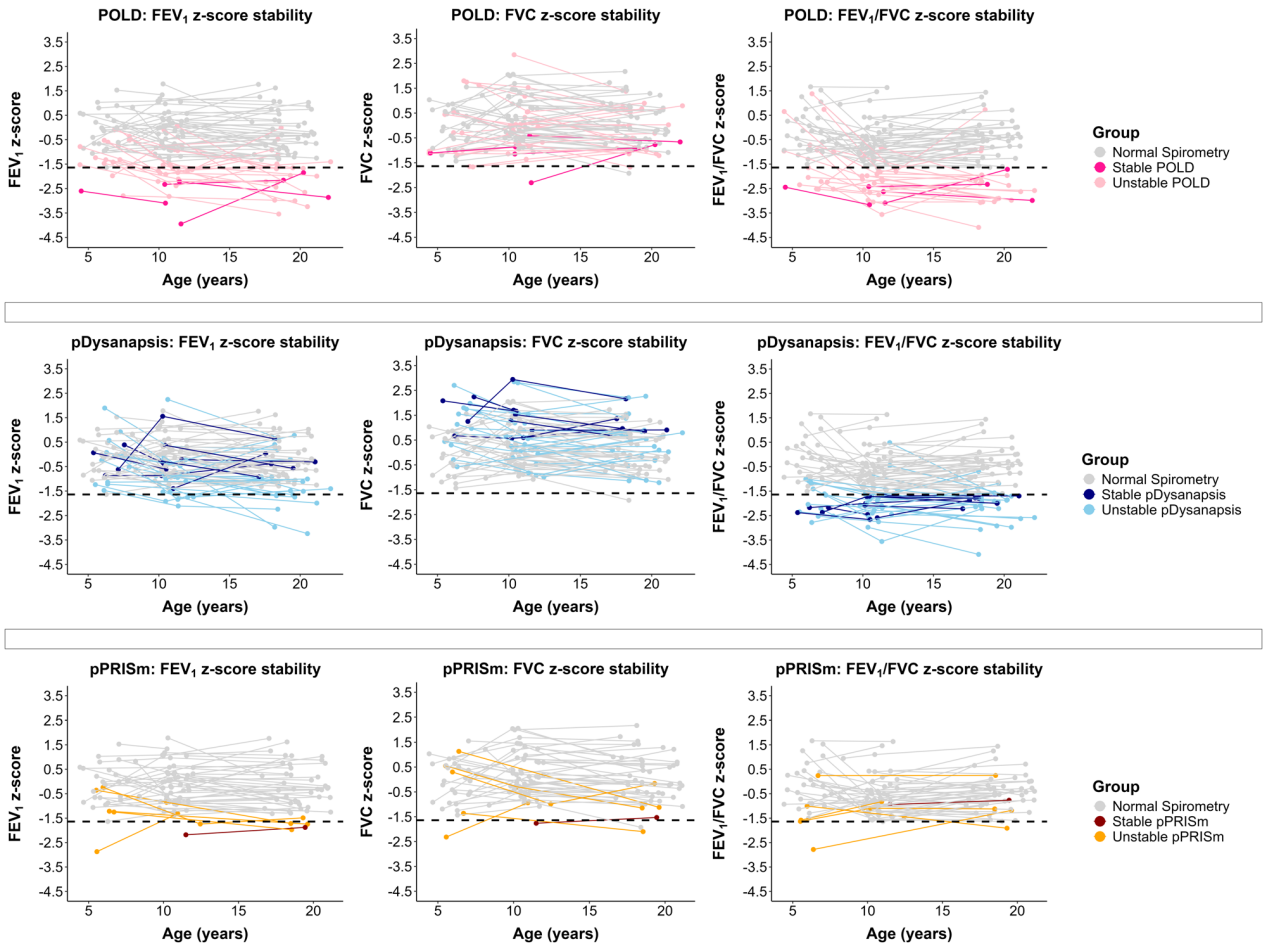
Instability of the spirometry phenotypes over serial lung function measurements. The dashed line represents the lower limit of normal. FEV_1_, forced expiratory volume in 1 s; FVC, forced vital capacity; POLD, prematurity-associated obstructive lung disease; pDysanapsis, dysanapsis of prematurity; pPRISm, prematurity-associated preserved ratio impaired spirometry.

Of those with ≥2 spirometry measurements, 36% (n=37) changed phenotypes. Of those who remained in the same phenotype, 78% (51/65) were classified with normal spirometry, 8% (5/65) with POLD, 2% (1/65) with pPRISm and 12% (8/65) with pDysanapsis. Of those who changed phenotypes, 51% (19/37) started as normal and changed to an abnormal phenotype and 5% (2/37) started as normal, changed to abnormal and then back to normal again, whereas 14% (5/37) started as abnormal phenotype and changed to normal spirometry and 30% (11/37) changed between abnormal phenotypes. Many of those that changed phenotype classification had a FEV_1_ or FEV_1_/FVC z-score sitting near the lower limits (figure 2). Of those with a neonatal diagnosis of BPD (n=63), a similar proportion changed phenotype classification compared with those that stayed in the same classification (41% (n=26) vs 59% (n=37), p=0.262).

## Discussion

The implementation of adult spirometry phenotypes to prematurity-associated lung disease reflects a growing effort to better characterise long-term lung function trajectories beyond the traditional bronchopulmonary dysplasia definition. Given the high symptom burden in this population, it is important to identify children with evidence of impairment that may be amenable to treatment. However, the instability of adult spirometry classifications across serial spirometry measurements suggests this approach does not adequately capture the complexity of prematurity-associated lung disease during the growth period.

We would expect that children born prematurely progress towards an obstructive phenotype as they age.^3 10 13^ Indeed, many children in this cohort progressed towards an abnormal phenotype. Other longitudinal studies have shown transitions between phenotypic classifications; in adult smokers, almost half of the individuals transitioned between PRISm and another lung function category over time,^14^ and population-based birth cohorts have shown that only a third of those with dysanapsis in childhood remained dysanaptic during adolescence.^15^

Within test variability for FEV_1_ has been shown to be ~5% in children and between test variability up to 20%^16^ suggesting that individuals with lung function measurements near the lower limits may be reclassified into an abnormal phenotype or to normal spirometry simply due to natural physiological variation in lung function rather than disease progression. The inter-test variability may also explain why some change from an abnormal phenotype to normal spirometry. Relying on spirometry alone, it remains unclear whether these changes reflect disease progression, resolution or simply the biological variability of the measurement. Spirometry may be insensitive to early lung changes.

The interpretation of our data is limited by the small numbers of individuals with complete data. Complete data at all three time points was constrained by the nature of recruitment, with some individuals not recruited until middle childhood, whereas others were lost to follow-up from early childhood through to adolescence. The differences in equipment used for time points 1 and 2 compared with time point 3 contribute to the variability. A limitation of our study is that our cohort may not be fully representative of the preterm population, being majority white (93%) from a metropolitan area in a high-income country, with those recruited being well enough and having socioeconomical advantage to enable participation in research. Those with major congenital abnormalities and significant neurodevelopmental disability that prevented them from completing pulmonary function testing were excluded from recruitment, which may overlook some of the sickest survivors of preterm birth. While these preliminary data are informative and hypothesis generating, data pooling initiatives, such as the Prematurity’s Effect on the Lungs In Children and Adults Network (PELICAN),^17^ will be invaluable for drawing more robust conclusions in the future. Further, acknowledging that our longitudinal cohort only spans the growth period, future studies in this group will help us understand if these phenotypes applied in early life have later prognostic value for COPD or pre-COPD.

## Conclusion

Applying adult spirometry labels does not address the complex heterogeneity and underlying mechanisms of the ongoing respiratory disease in this very preterm population. Our findings do stress the importance of structured long term follow-up of those born preterm.

## Data Availability

Data are available upon reasonable request.
